# C–C Bond formation catalyzed by natural gelatin and collagen proteins

**DOI:** 10.3762/bjoc.9.123

**Published:** 2013-06-07

**Authors:** Dennis Kühbeck, Basab Bijayi Dhar, Eva-Maria Schön, Carlos Cativiela, Vicente Gotor-Fernández, David Díaz Díaz

**Affiliations:** 1 Institut für Organische Chemie, Universität Regensburg, Universitätsstr. 31, Regensburg 93053, Germany, Tel: + 49 941 943 4681; Fax: + 49 941 943 4121; 2 CReST Unit, Chemical Engineering Div., National Chemical Laboratory, Dr. Homi Bhabha Road, Pune 411008, India; 3 Instituto de Síntesis Química y Catálisis Homogénea (ISQCH), Universidad de Zaragoza-CSIC, Pedro Cerbuna s/n, Zaragoza 50009, Spain; 4 Instituto Universitario de Biotecnología de Asturias, Universidad de Oviedo, Julián Clavería s/n, Oviedo 33071, Spain

**Keywords:** biocatalysis, carbon–carbon bond formation, gelatin, Henry reaction, protein

## Abstract

The activity of gelatin and collagen proteins towards C–C bond formation via Henry (nitroaldol) reaction between aldehydes and nitroalkanes is demonstrated for the first time. Among other variables, protein source, physical state and chemical modification influence product yield and kinetics, affording the nitroaldol products in both aqueous and organic media under mild conditions. Significantly, the scale-up of the process between 4-nitrobenzaldehyde and nitromethane is successfully achieved at 1 g scale and in good yield. A comparative kinetic study with other biocatalysts shows an increase of the first-order rate constant in the order chitosan < gelatin < bovine serum albumin (BSA) < collagen. The results of this study indicate that simple edible gelatin can promote C–C bond forming reactions under physiological conditions, which may have important implications from a metabolic perspective.

## Introduction

Gelatin is a mixture of hot-water-soluble proteins of high average molecular weights (50–100 kDa) derived primarily from collagen, which is the main naturally occurring structural protein in animal bones, skin and connective tissue (ca. one-third of the total protein mass in the body). Its low production cost and nontoxic, edible and biodegradable properties have made gelatin a common ingredient in food, pharmaceutical, cosmetic and photographic industries, among others [1]. There are two main types of gelatin: Type-A, with a broad iso-electric point (IEP) range of 6.0–9.0, and type-B, with a rather narrow IEP range of 4.5–5.5. The former results from the acidic hydrolysis of collagen, whereas the latter results from an alkaline treatment that causes a greater degree of deamidation of glutamine and asparagine.

Besides traditional applications in the food industry, gelatin has also gained attention in the scientific community as a reducing ligand and supporting media for the preparation of uniform metal nanoparticle catalysts [2–4]. In addition, the average composition of gelatin in terms of its amino acids content has been reported in several publications (arginine, glutamic acid, alanine, glycine, proline and hydroxyproline are the most abundant amino acids (ca. 10–25%)) [1], which makes the protein itself suitable for catalytic studies.

The Henry (nitroaldol) reaction is a versatile and widely used base-catalyzed C–C bond forming reaction between a nitroalkane and an electrophilic carbonyl derivative (aldehyde or ketone) to produce β-nitroalcohols, which can be transformed into valuable synthetic building blocks [5–10]. Some biopolymers such as salmon testes DNA [11] and chitosan [12], as well as various enzymes [13–16], have been reported to catalyze this type of reaction. However, to the best of our knowledge, the role of natural gelatin or collagen proteins as potential biocatalysts for C–C bond formation has not been yet described [17]. A practical importance of this study derives from the fact that gelatin is the protein most commonly associated with food products, in which different aldehydes may be also present. Therefore, the combination of this protein and aldehydes under physiological conditions could generate in vivo the formation of new metabolic products. Herein, we report for the first time the activity and comparative kinetics of gelatin and collagen proteins in the context of the Henry (nitroaldol) reaction.

## Results and Discussion

Reaction between 4-nitrobenzaldehyde (**1a**, 0.1 mmol) and nitromethane (**2a**) in DMSO was considered as the model reaction, observing that 2 mg of gelatin from porcine skin type-A (PSTA) catalyzed the selective formation of the corresponding nitroaldol product **3a** at physiological temperature. Thus, ca. 70% yield of **3a** was attained with 5-fold excess of **2a**, while higher loadings did not significantly improve the results (see Supporting Information File 1). Under these preoptimized conditions, further investigation of the solvent scope revealed DMSO as the most suitable organic solvent to carry out the reaction (Table 1, entries 1 and 2).

**Table 1:** Solvent-screening study for gelatin-mediated Henry reaction.^a^

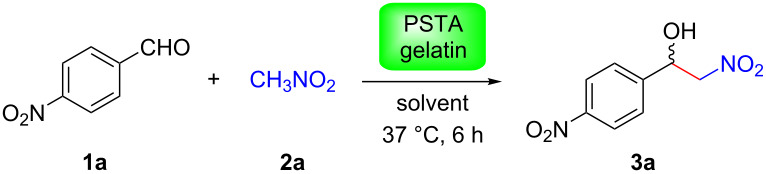		

Entry	Solvent	**3a**, Yield (%)^b^

1	EtOH, DMF, CH_3_CN or toluene	0 (0^c^)
2	DMSO	70 (0^c^)
3	H_2_O	14 (8^c^)
4	H_2_O/TBAB^d^	78 (8^c^)

^a^Reaction conditions: **1a** (0.1 mmol), **2a** (0.5 mmol), PSTA gelatin (2 mg), solvent (0.5 mL), 37 °C, 6 h. ^b^The ^1^H NMR yields correspond to the average values of two independent experiments (standard deviation, STDV = ± 2%). ^c^Control experiment: Reaction in the absence of gelatin. ^d^Tetra-*n*-butylammonium bromide (TBAB, 0.04 mmol). The addition of the phase transfer catalyst did not change the pH of the solution.

Interestingly, the reaction could be also performed in water, although in this case the addition of a phase transfer cocatalyst (e.g., TBAB) was necessary to achieve comparable results (Table 1, entries 3 and 4) [18]. In pure water, the yield could be also improved to ca. 60% by a 5-fold increase in the amount of gelatin. The observed beneficial effect of the phase-transfer catalyst also suggests potential relevance to physiological conditions, where biological membranes can be expected to serve a similar role. The background reaction in DMSO (i.e., control experiment in the absence of protein) was totally inhibited, and in H_2_O/TBAB represented only 10% of the product yield obtained in the presence of gelatin (entries 2 and 4), demonstrating the inherent catalytic activity of the protein under both organic and aqueous conditions.

In order to completely suppress the background contribution from the reaction media and concentrate on the pure effect of the protein catalyst, we continued our investigation with DMSO as the model solvent. It is also important to mention that the reaction can be scaled up with modest yields (i.e., **1a** (7.5 mmol), **2a** (37.5 mmol), PSTA gelatin (150 mg), DMSO (37.5 mL), 6 h, 37 °C, ca. 60% yield).

We also studied the effect of different types of gelatin obtained from various natural resources (i.e., PSTA, bovine skin type-B (BSTB) gelatin and cold-water fish skin (CWFS) gelatin). Comparable results were obtained in all cases (Table 2, entries 1–3), illustrating that properties such as IEP, polymer stiffness mass (Bloom number), and the extraction/recovery method used to isolate the protein (i.e., type-A, type-B) have no significant influence on the catalytic activity in DMSO. We also confirmed that there was no effect of possible metal impurities in the gelatin samples on the reaction conversion (see Supporting Information File 1). For further experiments, we used PSTA gelatin based on its lower price, known protein content, and slightly acidic pH value in solution.

**Table 2:** Influence of different types of gelatin in the model Henry reaction between **1a** and **2a** in DMSO.^a^

Entry	Gelatin type^b^	**3a**, Yield (%)^c^

1	PSTA gelatin	70
2	BSTB gelatin	75
3	CWFS gelatin	74
4	Succinylated PSTA gelatin	57^d^
5	Esterificated PSTA gelatin	27^d^
6	Powdered edible gelatin^e^	60^d^
7	Cooked sheet edible gelatin^f^	69^d^, 63^g^
8	PSTA gelatin hydrogel	33^h^ (33^i^, 2^j^)

^a^Reaction conditions: **1a** (0.1 mmol), **2a** (0.5 mmol), gelatin (2 mg), DMSO (0.5 mL), 37 °C, 6 h. ^b^See Supporting Information File 1 for preparation and experimental details. ^c^ The ^1^H NMR yields that correspond to the average values of two independent experiments (unless otherwise indicated, STDV = ± 2%). ^d^STDV = ± 5%. ^e^Purchased at the supermarket. ^f^Purchased at the supermarket and cooked for the experiment. ^g^Reaction carried out by using the xerogel material obtained from a cooked sheet of edible gelatin. ^h^Experiment performed at rt to preserve the gel phase of the catalyst obtained separately from 6 mg of PSTA gelatin in 0.3 mL of H_2_O. ^i^Control experiment: Reaction performed in a mixture DMSO (0.5 mL)/H_2_O (0.3 mL) and 6 mg of powdered PSTA gelatin (not gel phase). ^j^Control experiment: Reaction in DMSO (0.5 mL)/H_2_O (0.3 mL) without gelatin.

Moreover, chemical modification of the side chains of gelatin (i.e., succinylation, esterification) [19–20] suggested the importance of free carboxyl groups on the catalyst activity (e.g., Type A gelatin has ca. 78–80 millimoles of free carboxyl groups per 100 g of protein). The observations are in agreement with the examples reporting individual amino acids (e.g., alanine, proline) as catalysts for similar reactions [20–27]. Very interestingly, even the direct use of edible gelatin obtained from the supermarket (either in powdered or cooked form) promoted the C–C bond formation in good yields (Table 2, entries 6 and 7). On the other hand, although the use of gelatin in hydrogel form did not afford a higher yield than in solution under comparable conditions (Table 2, entry 8), the former provided a suitable way to work in a heterogeneous phase.

We further evaluated the possibility to convert different aldehydes in combination with nitromethane or nitroethane (Table 3). In general, aromatic aldehydes with strong or moderate electron-withdrawing substituents were easily converted into the corresponding nitroaldol products in moderate to very good yields over 6 h at 37 °C (Table 3, entries 1–6). These examples also demonstrated that the *ortho*- or *meta*-substituents did not hinder the reaction at all. In contrast, considerably lower yields were obtained in the cases of aromatic aldehydes bearing weak electron-withdrawing groups or electron-donating groups (Table 3, entries 8–12), while 2-pyridinecarbaldehyde led to 54% yield in the reaction with nitromethane (Table 3, entry 13). Aliphatic aldehydes (Table 3, entries 14 and 15) were poorly converted. However, these yields could be nearly doubled by increasing either the reaction time (e.g., 72 h) and/or the reaction temperature (e.g., 60 °C) (Table 3, entries 7, 9, 11, 12 and 14). Importantly, control experiments in the absence of gelatin at 60 °C also showed no product formation (Table 3, entries 1 and 7). It is important to remark that even vanillin or citronellal, which are also components of many foods, could be partially converted to nitroaldol products by gelatin (Table 3, entries 12 and 15). When nitroethane (pK_a_ = 8.6) was used as the nucleophile instead of nitromethane (pK_a_ = 10.2) the yield increased considerably (Table 3, entries 2, 6, 10 vs. 1, 5, 9, respectively), albeit without significant diastereoselectivity. Thus, acidity of the nitroalkane plays here a more important role than steric effects [28]. It is worth mentioning that control experiments in the absence of gelatin with nitroethane also provided a much lower conversion than in the presence of the protein (Table 3, entry 2), although it was significant in comparison to the less reactive nitromethane (Table 3, entry 1).

**Table 3:** Substrate scope of the gelatin-catalyzed Henry reaction in DMSO.^a^

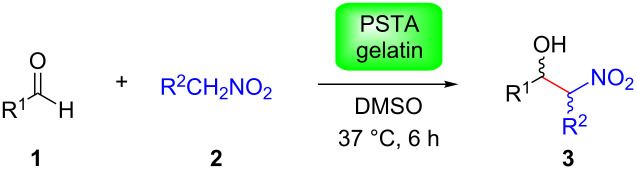				

Entry	R^1^ (**1**)^b^	R^2^ (**2**)	**3**, Yield (%)^c^	dr^d^

1	(4-NO_2_)-C_6_H_4_	H	70 (0^e^)	NA
2	(4-NO_2_)-C_6_H_4_	CH_3_	84 (44^e^)	1.1:1
3	(2-NO_2_)-C_6_H_4_	H	77	NA
4	(3-NO_2_)-C_6_H_4_	H	90	NA
5	(4-NC)-C_6_H_4_	H	78	NA
6	(4-NC)-C_6_H_4_	CH_3_	92	1.1:1
7	C_6_H_5_	H	5 (11^f^, 10^g^, 0^h^)	NA
8	(4-Br)-C_6_H_4_	H	24	NA
9	(4-Cl)-C_6_H_4_	H	13 (22^f^)	NA
10	(4-Cl)-C_6_H_4_	CH_3_	39	1.2:1
11	(4-CH_3_)-C_6_H_4_	H	4 (6^f^)	NA
12	(4-OH, 3-CH_3_O)-C_6_H_3_	H	6^i^ (24^f,i^)	NA
13	Pyrid-2-yl	H	54	NA
14	(CH_3_)_2_CHCH_2_	H	8 (13^f^)	NA
15	(CH_3_)_2_C(CH_2_)_2_CH(CH_3_)CH_2_	H	6^j^	NA

^a^Reaction conditions: **1** (0.1 mmol), **2** (0.5 mmol), PSTA gelatin (2 mg), DMSO (0.5 mL), 37 °C, 6 h. ^b^See Supporting Information File 1 for expanded structures. ^c^The ^1^H NMR yields that correspond to the average values of two independent experiments (standard deviation, STDV = ± 2%). ^d^Diastereomeric ratio (*anti/syn*) determined by ^1^H NMR analysis. Relative configurations were assigned by comparison with data in the literature. NA = Not applicable. ^e^Control experiment made in the absence of gelatin. Reaction time = 6 h, temperature = 37 °C. ^f^Reaction time = 72 h, temperature = 37 °C. ^g^Reaction time = 6 h, temperature = 60 °C. ^h^Control experiment made in the absence of gelatin. Reaction time = 6 h, temperature = 60 °C. ^i^Yield of β-nitroalkene. ^j^Yield of dinitroalkane. In this case, β-nitroalcohol was also identified in trace amounts.

**Figure 1:**
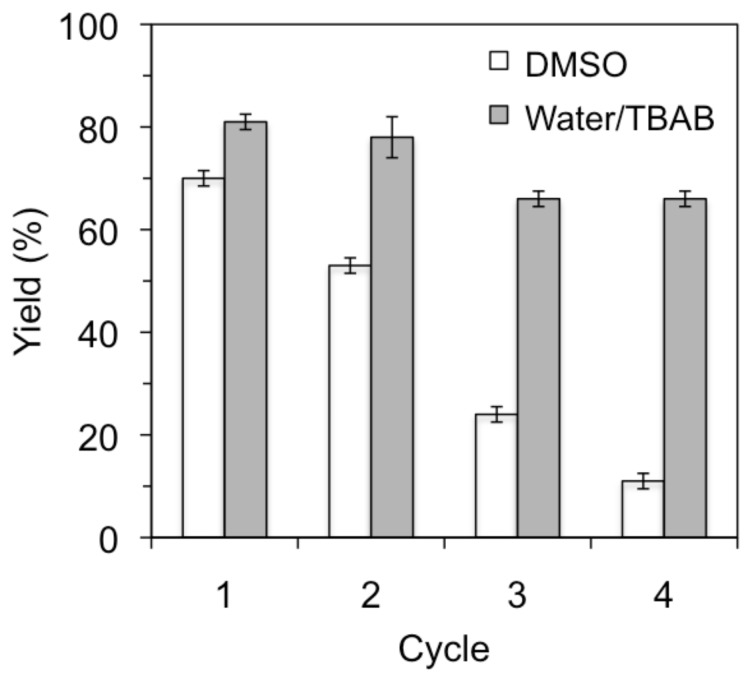
Typical recycling experiments for the gelatin-catalyzed Henry reaction. Reaction conditions: 4-Nitrobenzaldehyde (**1a**, 15.1 mg, 0.1 mmol), nitromethane (**2a**, 27 μL, 0.5 mmol), solvent (0.5 mL), PSTA gelatin (2 mg), 37 °C, 6 h. Yields correspond to ^1^H NMR values obtained from at least three independent experiments. For the experiments in water/TBAB, additional TBAB was added after each cycle (i.e., 2.6 mg after 1st cycle, 5.2 mg after 2nd cycle, 2.4 mg after 3rd cycle. These quantities corresponded to the loss of TBAB after each cycle as determined by ^1^H NMR analysis).

On the other hand, the gelatin catalyst could be recovered and reused for further cycles, albeit unfortunately with gradual deactivation of the catalyst in both organic and aqueous media (Figure 1). This result may be associated with (1) gradual loss of catalyst loading between cycles and/or (2) possible formation of linear or cyclic aminals [12]. Interestingly, when water/TBAB was used as the solvent system the reduction of the catalytic activity was less dramatic than in the case of DMSO. However, such apparently better performance in water/TBAB was dependent on the addition of extra TBAB after each cycle in order to ensure a constant concentration of the phase-transfer catalyst during the reaction. The continuing loss of TBAB during the work-up after each cycle was quantified by ^1^H NMR analysis of the reaction crude.

Very interestingly, we found that the direct use of the precursor collagen as biocatalyst also afforded the desired product in very good yields. In this case, the most common motifs in the amino acid sequence, which could be also associated with catalytic sites, are glycine-proline-X and glycine-X-hydroxyproline, where X is any other amino acid (see Supporting Information File 1). However, despite gelatin and collagen forming triple helices as a chiral secondary structure, HPLC analysis of the reaction mixtures revealed negligible enantioselectivity. This lack of selectivity is in agreement with previous publications dealing with other biocatalysts used in the Henry (nitroaldol) reaction [11–16].

**Figure 2:**
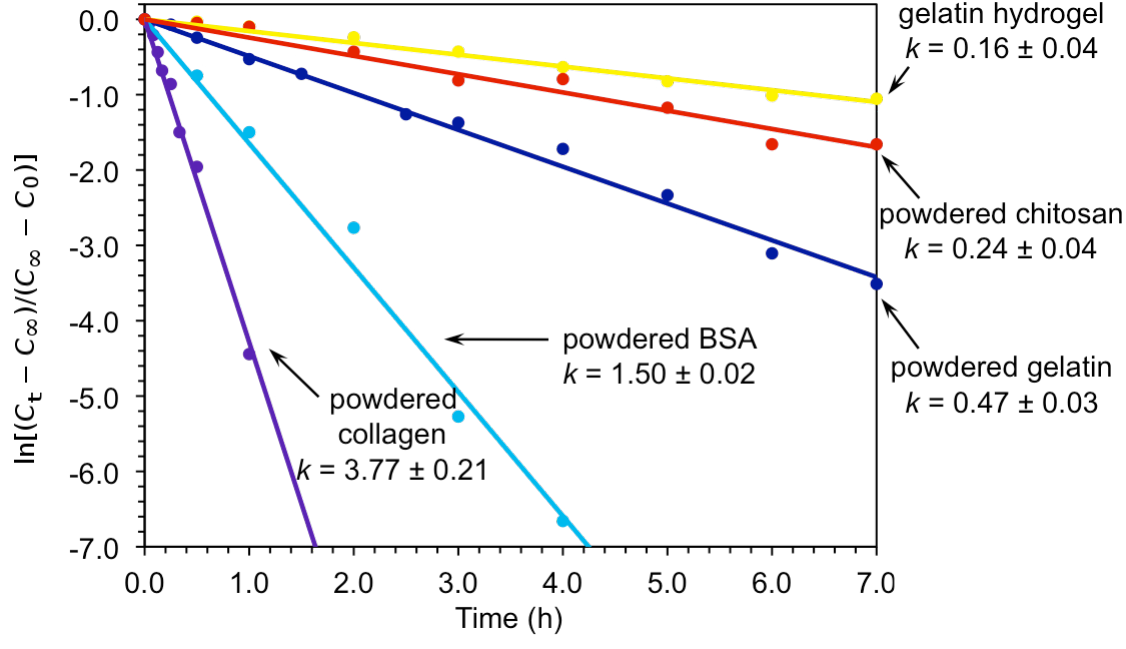
First-order kinetics plots for the model Henry reaction between **1a** and **2a** catalyzed by different systems. Apparent rate constants are in units of h^−1^. Each data point represents the average of two independent measurements. *C*_∞_ = final concentration at infinite time; *C*_t_ = concentration at given time *t*; *C*_0_ = initial concentration at *t* = zero time.

At this point, and in order to draw a meaningful comparison with other known biocatalytic systems (i.e., chitosan [29], bovine serum albumin (BSA) [30–31]) we carried out the kinetic analysis of the model reaction for each case [8]. Figure 2 shows the first-order kinetics plots demonstrating the fine-tuning of the reaction rate in response to the biocatalyst used. In the case of biopolymers in powder form, first-order rate constants increased in the order chitosan < gelatin < BSA < collagen, whereas the same concentration of gelatin in hydrogel form showed slower kinetics. These results suggest a detrimental decrease of accessibility to the active groups of the catalyst upon gel formation. Nevertheless, it should also be noted that creating, for example, aerogels of the corresponding powdered materials (e.g., chitosan) would significantly enhance their reactive surfaces and, therefore, their catalytic performance.

**Figure 3:**
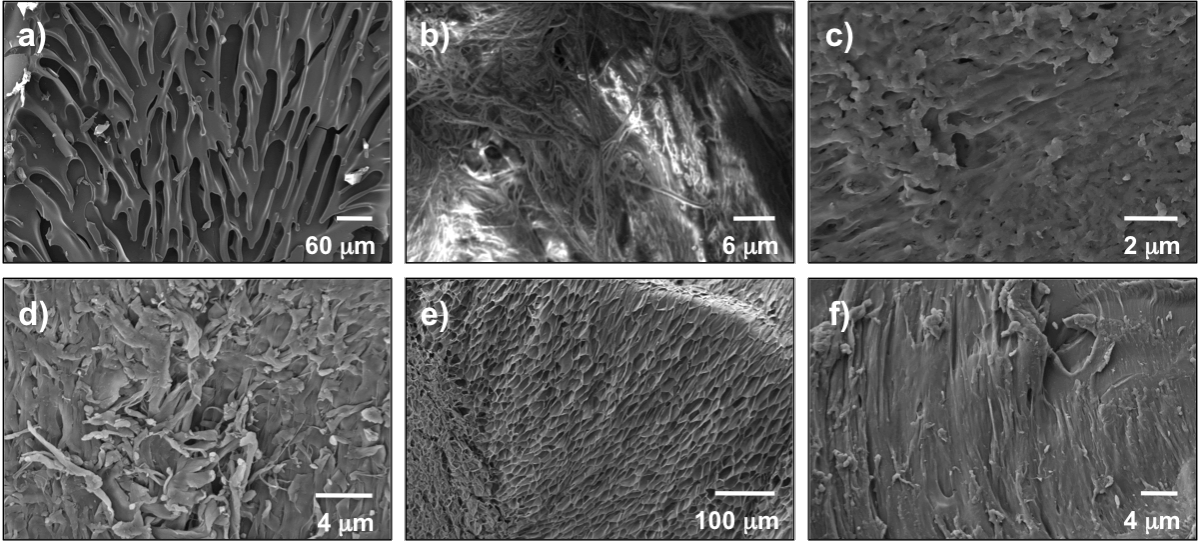
Selected FESEM images of different catalysts used for comparative kinetics: (a) powdered BSA; (b) powdered collagen; (c) powdered PSTA gelatin; (d) powdered chitosan; (e) xerogel prepared by freeze-drying the hydrogel made of PSTA gelatin; (f) commercial powdered edible gelatin.

Moreover, field-emission scanning electron microscopy (FESEM) images of the biocatalysts associated faster reactions with porous fibrilar morphologies and slower kinetics with thicker and close-grained surfaces (Figure 3). These results suggest that the morphology and/or physical state of the proteins play an important role in the kinetics of the nitroaldol reaction.

## Conclusion

In conclusion, we have found that natural gelatin and collagen proteins are able to promote C–C bond formation via the Henry (nitroaldol) reaction between various aldehydes and nitroalkanes. Thus, the reaction takes place in both aqueous and organic media under mild conditions, affording the nitroaldol product in variable yields depending on the aldehyde and nitroalkane nature. Moreover, the scale-up of the process between 4-nitrobenzaldehyde and nitromethane could also be achieved on a 1 g scale and in good yield. A comparative kinetics study with other biocatalysts showed an increase of the first-order rate constant as follows: Chitosan < gelatin < BSA < collagen. Remarkably, the morphology and the physical state of the protein play an important role on the kinetics of the nitroaldol reaction. It should be emphasized that although none of the biopolymers are superior to standard base catalysts, such as tetramethylethylenediamine [8], from a synthetic point of view, the former avoided byproduct formation and offered the possibility to work under advantageous ecofriendly, sustainable and heterogeneous conditions. However, it is indeed more important to realize that edible gelatin or collagen have the potential to partially convert aldehydes that are usually present in numerous foods or cosmetics, under physiological conditions, which could modify their metabolic routes.

## Experimental

^1^H NMR spectra were recorded at 25 °C on a Bruker Avance 300 spectrometer. Chemical shifts are denoted in δ (ppm) relative to tetramethylsilane (TMS δ = 0) as an internal standard or relative to residual solvent peaks. Samples were analyzed by chiral-phase HPLC using a Varian 920-LC Liquid Chromatograph and a column Phenomenex Lux Cellulose-1, 4.6 × 250 mm, 5 μm. TLC was facilitated by the use of the following stains in addition to UV light (254 nm) with fluorescent-indicating plates (aluminium sheets precoated with silica gel 60 F254, Merck): phosphomolybdic acid, vanillin, iodine.

Analytical-grade solvents and commercially available reagents were purchased from TCI Europe or Sigma Aldrich and were used as received. Gelatin Porcine skin type A (PSTA) (Cat. No. G2500-100G; Batch No. 128K0066; CAS 9000-70-8; Type A, derived from acid-cured tissue; ~300 Bloom; 79% protein content by Biuret), gelatin bovine skin type B (Cat. No. G9382-100G; Lot No. 051M0012V; CAS 9000-70-8; Type B, derived from lime-cured tissue; ~225 Bloom; 73% protein content by Biuret) and gelatin from cold water fish skin (Cat. No. G7041-100G; Lot No. 071M0258V; CAS 9000-70-8; ash content 0.3%; heavy metals content 1 ppm; viscosity 8.9 CS, 10% solution, 30 °C) were purchased from Sigma Aldrich and used without further purification. Modification of side chains of gelatin, succinylation and esterification reactions of carboxylic groups of PSTA gelatin were carried out as previously described.

The catalyst samples were observed with a Carl Zeiss Merlin field-emission scanning electron microscope (FESEM, resolution 0.8 nm) equipped with a digital camera and operating at 5 kV (accelerating voltage) and 10 μA (emission current). Xerogel samples of the corresponding hydrogels were prepared by the freeze-drying (FD) method [32]. The resulting material was placed on top of a tin plate and shielded by Pt (40 mA during 30 s for FE-SEM; film thickness ≈5 nm).

### Typical procedure for gelatin-catalyzed Henry reaction

Nitromethane (27 μL, 0.5 mmol) was added in one portion to a 4 mL screw cap vial containing 4-nitrobenzaldehyde (15.1 mg, 0.1 mmol), PSTA gelatin (2 mg) and DMSO (0.5 mL). The mixture was stirred (250 rpm) for the appropriate time at 37 °C. The reaction was quenched by the addition of EtOAc (1 mL) and EtOH (1 mL) and subsequent filtration of the precipitated catalyst. The filtrate was rinsed three times with EtOAc (1 mL), and the combined organic phases were washed with H_2_O (2 × 5 mL) and brine (5 mL), dried over Na_2_SO_4_, filtered and evaporated under reduced pressure to afford the crude product.

Yield was determined by ^1^H NMR of the crude product in CDCl_3_ using diphenylmethane (1 mL of a 0.1 M stock solution) as the internal standard. The result was confirmed by a second experiment using directly dimethylacetamide (9.2 μL, added using a Hamilton syringe) as the internal standard. Thus, possible concentration variations of the stock solution of diphenylmethane in CDCl_3_ could be detected and the values crosschecked. In the case of diphenylmethane, three different methods to introduce the standard were evaluated: (A) Introduction of the standard from the stock solution in CDCl_3_ after complete work-up of the reaction; (B) internal standard was present in the mixture during the reaction and work-up; (C) internal standard was introduced into the reaction mixture before the work-up. The yields obtained in the above method by using the model reaction between **1a** (0.1 mmol), **2a** (0.5 mmol), DMSO (0.5 mL), and PSTA gelatin (2 mg), at 37 °C, for 6 h, were (A) = 70%; (B) = 2%; (C) 76%. In all further experiments, we used method (A) to quantify the ^1^H NMR yield.

### Typical procedure for gelatin hydrogel-catalyzed Henry reaction

A mixture of PSTA (6 mg) in H_2_O (0.3 mL) was gently heated in a sealed screw cap vial (4 mL) until a homogeneous solution was obtained. This solution was stored overnight at rt to promote gel formation, which was confirmed by the complete absence of gravitational flow upon turning the vial upside down. Then, a solution consisting of 4-nitrobenzaldehyde (15.1 mg, 0.1 mmol) and nitromethane (27 μL, 0.5 mmol,) in DMSO (0.5 mL) was added on top of the gel. The vial was stored without shaking for 24 h at rt to allow diffusion. After this time, EtOAc (1 mL) and EtOH (1 mL) were added to quench the reaction and remove the supernatant organic layer. Next, the gel was gently heated to obtain a solution, which was further diluted with H_2_O (2 mL) and EtOAc (2 mL), and finally extracted with EtOAc (2 × 2 mL). The combined organic layers were dried over anhydrous Na_2_SO_4_, filtered and evaporated under reduced pressure to obtain the crude product. The ^1^H NMR yield was determined as described above.

### Notes

a) A control experiment to quantify any possible effect of the hydrogel on the reaction was carried out as follows: PSTA (6 mg), 4-nitrobenzaldehyde (15.1 mg, 0.1 mmol), nitromethane (27 mL, 0.5 mmol,) H_2_O (0.3 mL) and DMSO (0.5 mL) were mixed in a screw cap vial (4 mL) and the mixture was stirred for 24 h at rt. After this time, EtOAc (1 mL) and EtOH (1 mL) were added to quench the reaction. The mixture was diluted with H_2_O (2 mL) and EtOAc (2 mL), and finally extracted with EtOAc (2 × 2 mL). The combined organic layers were dried over anhydrous Na_2_SO_4_, filtered and evaporated under reduced pressure to obtain the crude product. The ^1^H NMR yield was determined as described above.

b) For the reaction with cooked gelatin purchased from the supermarket, 10 g of gelatin sheets were dissolved in 100 mL water by heating it on a heating plate, and the mixture was stored overnight in the fridge. The reactions were carried out with 20 mg of the formed hydrogel.

c) All condensation products are known and the spectroscopic data obtained from the NMR analysis of the reaction mixtures were in agreement with those reported in the literature (see Supporting Information File 1).

### Recycling experiments

In general, acetone or ethanol (1 mL per 2 mg of catalyst) could be used to precipitate all of the gelatin catalyst, which could be further separated by centrifugation (10 min, 3800 rpm), washing with EtOAc (2 mL), centrifugation cycles, and finally drying of the residue under vacuum before the next catalytic cycle. Additionally, direct extraction of aqueous solutions with EtOAc may allow reuse of the aqueous solution of the catalyst in subsequent cycles.

### Kinetics studies

Reaction conversions were unambiguously calculated by ^1^H NMR analysis of the reaction mixtures according to the integration of the characteristic signals of the species in the reaction mixture in the presence of an appropriate internal standard. Each experimental point represents the average of at least two independent experiments. Among various kinetics models, lines presented in the kinetics plots show best-fits of the first-order model for each case (i.e., [NO_2_R] ≥ [aldehyde]). Nevertheless, the possibility of more complex kinetics was also suggested in some cases where the fits were not ideal (e.g., TMEDA: presence of a fast introductory phase and subsequent stagnation of the reaction rate: *t* = 30 s, yield 52%; *t* = 60 s, yield 60%; *t* = 90 s, yield 66%; *t* = 120 s, yield 67%; *t* = 180 s, yield 63%; *t* = 240 s, yield 67% (Figure S3) [8]). Due to the fact that not all reactions reached 100% yield, data fitting was made according to the variation of ln[(*C*_t_ − *C*_∞_)/(*C*_∞_ − *C*_0_)] with time, where *C*_t_ is the concentration at a given time *t*; *C*_∞_ the final concentration (at infinite time) and *C*_0_ the initial concentration (at *t* = zero time). For reaction conversions close to 100%, plots of ln(*C*_t_/*C*_0_) versus time provided consistent results (*C*_∞_ = 0). Under these considerations, minor differences were observed between the exponential and linear fits. All errors reported for the rate constants *k* were calculated by graphical analysis. Solubility of gelatin during the reaction was found to play no role in the product yield.

## Supporting Information

File 1 Optimization studies, additional experiments, figures and tables.
