# Supplementary material for: C–C Bond formation catalyzed by natural gelatin and collagen proteins
Source: Beilstein J Org Chem. 2013 Jun 7;9:1111–8. doi: 10.3762/bjoc.9.123 (PMC3701417; doi:10.3762/bjoc.9.123)
Supplement: File 1 — Optimization studies, additional experiments, figures and tables. [file Beilstein_J_Org_Chem-09-1111-s001.pdf]

Supporting Information

for

C–C Bond formation catalyzed by natural gelatin and collagen proteins

Dennis Kühbeck[1], Basab Bijayi Dhar[2], Eva-Maria Schön[1], Carlos Cativiela[3], Vicente Gotor-Fernández[4] and David Díaz Díaz*[,1,3]

[1]Institut für Organische Chemie, Universität Regensburg, Universitätsstr. 31, Regensburg 93053, Germany, Tel: + 49 941 943 4681; Fax: + 49 941 943 4121, [2]CReST Unit, Chemical Engineering Div., National Chemical Laboratory, Dr. Homi Bhabha Road, Pune 411008, India, [3]Instituto de Síntesis Química y Catálisis Homogénea (ISQCH), Universidad de Zaragoza-CSIC, Pedro Cerbuna s/n, Zaragoza 50009, Spain and [4]Instituto Universitario de Biotecnología de Asturias, Universidad de Oviedo, Julián Clavería s/n, Oviedo 33071, Spain.

Email: David Díaz Díaz - David.Diaz@chemie.uni-regensburg.de

*Corresponding author

Optimization studies, additional experiments, figures and tables.

Contents list

		page
1.	Expanded introduction …….…………………..	S2
2.	Preliminary optimization experiments …….………………………………	S3
3.	Additional experiments …….………………………………………………	S4
4.	Kinetics studies ……………………………………………………...............	S5
5.	Representative ^{1}H NMR spectra …….…………………………..……………	S6
6.	Additional FESEM images ………………………………………………….	S7
7.	References ……………………………………………………………...........	S7

1. Expanded introduction

• Henry reaction

The Henry (nitroaldol) reaction, discovered more than 100 years ago [1], is one of the most important C–C bond-forming reactions used to generate β-nitroalcohols, which constitute versatile organic synthons for the synthesis of nitroalkenes, 2-nitroketones, 2-aminoalcohols, α-hydroxy carboxylic acids and bioactive compounds including, among others, antibiotics, fungicides and insecticides [2]. Due to its practical importance, a vast number of publications have focused on the development of new catalysts for this transformation, including amines, alkali-metal bases, ammonium salts, guanidine derivatives, organo-phosphorous derivatives, Lewis acids or electrophilic activators [3].

Nevertheless, the major limitation of conventional catalysis has been the formation of several byproducts, including those originated from aldol self-condensation, polymerization, Cannizzaro and water-elimination reactions. On the other hand, the reaction usually requires organic solvents, a large excess of nitroalkanes, long reaction times, and challenging time- and labor-intensive isolation techniques [4]. Hence, the development of new sustainable, eco-friendly and selective catalysts for C–C bond formation also in aqueous media continues to be an important task [5]. In this sense, microwave techniques, solvent-free conditions and biocatalytic processes represent attractive alternatives [3,6]. In addition, substituent- and catalyst-dependent selectivity toward aldol or nitrostyrene products in a heterogeneous base-catalyzed Henry reaction has been also reported [7].

• Gelatin and collagen features

Although gelatin has been known to mankind for over 3000 years, it continues to find new uses in additional research areas, as we have demonstrated in this paper. Looking at its properties, gelatin has a molecular weight distribution between 50–100 kD. Type-A gelatin has ca. 78–80 millimoles of free carboxyl groups per 100 g of protein and a broad iso-electric point (IEP) [8] between 6.0–9.0. In contrast, type-B gelatin has 100–115 millimoles of free carboxyl groups per 100 g of protein and a rather narrow IEP range of 4.5–5.5.

Table S1: Approximate amino acid composition of gelatin.

Entry	Aminoacid	Content (%)
1	Tyrosine	0.4
2	Methionine	0.7
3	Histidine	0.8
4	Hydroxylysine	0.9
5	Isoleucine	1.0
6	Phenylalanine	1.1
7	Threonine	2.0
8	Valine	2.2
9	Leucine	2.5
10	Lysine	2.9
11	Serine	3.4
12	Aspartic acid	4.8
13	Arginine	10.0
14	Glutamic acid	10.3
15	Alanine	10.4
16	Hyroxyproline	11.4
17	Proline	12.0
18	Glycine	23.2

Moreover, type-A gelatin is derived from acid-cured tissue, whereas type-B gelatin is derived from lime-cured tissue. Protein content, amino acid composition and IEP differ depending on the gelatin source and extraction method. Thus, protein content in PSTA gelatin is 79% (pH value for 1.5% solution at 25 °C = 3.8–5.5), whereas in BSTB gelatin is 73% (pH value for 1.5% solution at 25°C = 5.0–7.5) [9]. Edible gelatin purchased at the supermarket has 83%

protein content [10]. Table S1 outlines the general amino acid composition of gelatin, sorted in increasing content [11]. Several of these amino acids have been proven to be active in amine-based catalysis, including the Henry reaction. Thus, gelatin seems to be a natural choice as a polyvalent catalyst for this reaction.

On the other hand, gelatin forms thermoreversible hydrogels in water, with melting temperatures below body temperature (< 35 °C). From a rheological point of view, both the storage and loss shear modulus G' and G'' exhibit a power-law dependence on the frequency at the sol-gel transition point (rheological exponent $n = 0.58 \pm 0.05$) [12].

For potential alternative experiments dealing with gelatin catalysis, it should be also considered that gelatin proteins in aqueous media are prone to degradation on incubation at elevated temperatures (> 40–50 °C), where the salt content has an influence on both the unfolding of the polypeptide chains and the hydrolytic stability [13,14]. In addition, gelatin hydrogel (prepared without any additional hardener) was found to be fairly stable at rt in water, hexanes, Et_2O, EtOAc and cyclohexane, but undergoes shrinking and clear-to-opaque transition in MeCN, THF, acetone and MeOH. Clear-to-opaque transition, but no shrinking, was observed in Et_3N, CH_2Cl_2 and $CHCl_3$.

In the case of collagen, triple-stranded helical collagen molecules are stabilized by numerous hydrogen bonds and further laterally associated to form collagen fibrils of 10–300 nm diameter. Figure S1 shows the typical procedure for the extraction of different types of gelatin from collagen.

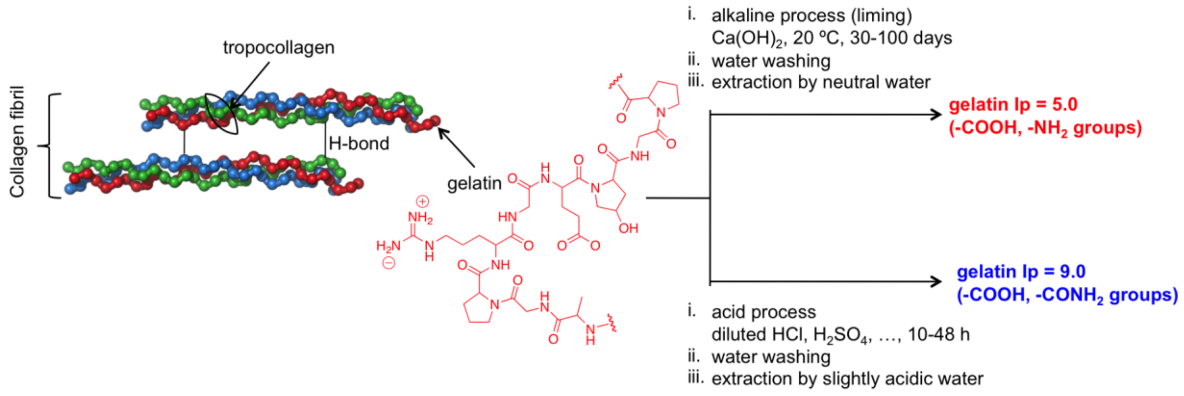

Figure S1: Representative preparative process for acidic and basic gelatins from collagen. A typical structure of gelatin is –Ala-Gly-Pro-Arg-Gly-Glu-4Hyp-Gly-Pro-.

• Possible metabolic implications

It is remarkable the fact that edible gelatin can promote C−C bond forming reactions under physiological conditions. In this regard, there are numerous aldehydes that are also present in a variety of foods (as well as cosmetic products) that could be, at least partially, transformed in the presence of gelatin proteins. Such process could influence to some extent the metabolic route of these compounds. In this sense, relevant aldehydes include, among others, (a) citronellal (the main component in the mixture of terpenoid compounds that provides citronella oil its distinctive lemon scent. It is isolated from lemon-scented gum and teatree), (b) citral or lemonal (present in the oil of several lemon plants widely used as a flavoring in the food industry and as an antimicrobial), (c) cinnamaldehyde (present in the bark of cinnamon trees and provides cinnamon its flavor and odor. It is widely use as a flavoring agent, agrochemical, antimicrobial and anticancer agent), (d) safranal (isolated from saffron and primarily responsible for this aroma in foods).

2. Preliminary optimization experiments

Preliminary screening of the reaction conditions by using 4-nitrobenzaldehyde (**1a**, 0.1 mmol) and nitromethane (**2a**) as a model reaction showed that 2 mg of PSTA gelatin afforded the corresponding nitroaldol product in good yields within 6 h at physiological temperature by

using at least a 5-fold excess of **2a** (Tables S2−S3).

For solvent screening, see discussion in the main paper.

Table S2: Screening of nitromethane equivalents.[a]

Entry	$MeNO_2$ (equivalents)	Time (h)	Yield (%)[b]
1	1	5	30
2	2	6	39
3	5	6	68
4	10	5	69

[a] Conditions: 4-Nitrobenzaldehyde (15.1 mg, 0.1 mmol), DMSO (0.5 mL), PSTA gelatin (2 mg), at physiological temperature. [b] ^{1}H NMR yield was determined with diphenylmethane as internal standard.

Table S3: Screening of catalyst loading.[a]

Entry	Temperature (°C)	Time (h)	Catalyst loading (mg)	Yield (%)[b]
1	25	19	0	0
2	25	19	5	62
3	25	7	10	63
4	25	5	15	54
5	40	5	0	0
6	40	5	2	66
7	40	2.5	5	72

[a] Conditions: 4-Nitrobenzaldehyde (15.1 mg, 0.1 mmol), nitromethane (54 μL, 1.0 mmol), DMSO (0.5 mL), PSTA gelatin (2 mg). [b] ^{1}H NMR yield was determined with diphenylmethane as internal standard.

Table S4: Screening of catalyst loading and effect of solvent.[a]

Entry	Solvent	Temperature (°C)	Time (h)	Catalyst loading (mg)	Yield (%)[b]
1	DMSO	37	6	2	70
2	DMSO	37	6	12	73
3	H_2O	37	6	2	14
4	H_2O	37	6	12	55

[a] Conditions: 4-Nitrobenzaldehyde (0.1 mmol), nitromethane (0.5 mmol), solvent (0.5 mL). [b] ^{1}H NMR yield was determined with diphenylmethane as the internal standard.

Note that regarding the catalyst loading; the use of more than 10 mg of gelatin may make the work-up of the reaction more laborious.

3. Additional experiments

a) We carried out a series of experiments that confirmed no effect of possible metal impurities in the gelatin samples on the reaction conversion. Possible impurities of gelatin samples are moisture content (ca. 10%), heavy metals (e.g., Co, Cu, Fe, Ni, Pd) and ash [15]. Table S5 shows the results of this study.

Note that the direct use of nondiluted metal stock solutions (these are 100-fold more concentrated than in the experiments according to the published values [16]) yielded the β-nitroalcohol product in only 2%.

Table S5: Effect of possible heavy metals in gelatin samples on the Henry reaction.[a]

Entry	Co(II) (10^{-6} mol)	Cu(II) (10^{-6} mol)	Fe(II) (10^{-6} mol)	Ni(II) (10^{-6} mol)[b]	Yield (%)[b]
1	1.98	7.8	1.0	1.88	1
2	198	78	100	188	2

[a] Conditions: 4-Nitrobenzaldehyde (0.1 mmol), nitromethane (0.5 mmol), DMSO (0.5 mL), 37 °C, 6 h. Metals were introduced into the reaction mixture from their corresponding stock solutions at the concentration reported in the literature to be present in gelatin samples (entry 1). [b] ^{1}H NMR yield determined with diphenylmethane as the internal standard.

b) The contribution of the pH of the media was investigated by individual measurements

(total volume of each sample = 10 mL) for the reaction between **1a** and **2a** in H_2O/TBAB at 37 °C and concentrations as described in the main text: milliQ water: pH 7.02; **2a** in milliQ water: pH 5.63; **1a** in milliQ water: pH 4.89; PSTA gelatin in milliQ water: pH 5.31; TBAB in milliQ water: pH 6.11; all 4 components combined in milliQ water: pH 4.36; **1a** + **2a** in milliQ water: pH 3.44; **1a** + **2a** + PSTA gelatin in milliQ water: pH 3.80.

c) Only in the cases of less reactive aldehydes, such as vanillin or iso-valeraldehyde, was it possible to detect by ^{1}H NMR also the formation of additional by-products (compounds **4**–**5**, Figure S2), albeit in trace amounts (≤ 2%). In this sense, β-nitroalkene **4** seemed to be the major product formed in the case of vanillin, whereas formation of β-nitroalcohol **3** was evident in the case of iso-valeraldehyde. However, the complexity of the ^{1}H NMR spectra in these cases made difficult the unambiguous assignment of all signals.

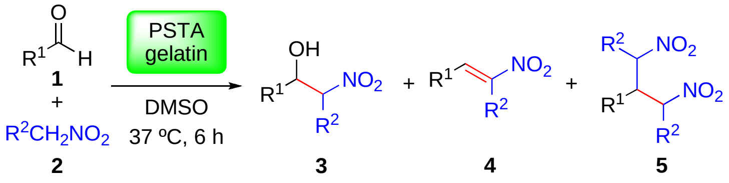

Figure S2: Henry reaction showing possible formation of β-nitroalcohol, β-nitroalkene and dinitroderivative in the case of some aldehydes as mentioned above.

d) Control experiments in the absence of gelatin were also performed with different aldehydes (*e.g.*, benzaldehyde) showing no conversion in DMSO as solvent, even at 60 ºC during 6 h.

e) Other heterocyclic aldehydes such as furfural were converted into the corresponding nitroaldol product in very low yield (ca. 4%). However, it should be considered that clean synthesis of such heteroarylnitroalcohols may require lower reaction temperatures [16]. Experiments in this regard were not pursued. Here, the use of a mixture DMSO:H_2O 4:1 as solvent, and benzaldehyde as substrate, did not show significant improvement in comparison to pure DMSO. In MeOH:H_2O 1:1 or MeCN:H_2O 1:1, no reaction product was observed.

f) Both air-dried and freeze-dried (lyophilized) xerogel materials prepared from the corresponding hydrogels provided similar results in the model reaction. Hence, potential water uptake of the xerogel specimen did not play any critical role in the reaction.

4. Kinetics studies

Table S6: Representative kinetic study in tabular form for the gelatin-catalyzed Henry reaction.[a]

Entry	Time (h)	Yield (%)[b]	$c_{product}$ (mol L^{-1})
**1**	0.25	5 ± 0.7	0.01 ± 0.001
**2**	0.5	15 ± 0.7	0.03 ± 0.001
**3**	1.0	28 ± 0.7	0.06 ± 0.001
**4**	1.5	35 ± 6.4	0.07 ± 0.013
**5**	2.0	51 ± 0.0	0.10 ± 0.000
**6**	2.5	48 ± n.d.	0.10 ± n.d.
**7**	3.0	50 ± 1.4	0.10 ± 0.003
**8**	4.0	56 ± 3.5	0.11 ± 0.007
**9**	5.0	60 ± 0.7	0.12 ± 0.001
**10**	6.0	70 ± 1.4	0.14 ± 0.003
**11**	18.0	62 ± n.d.	n.d.

[a] Conditions: 4-Nitrobenzaldehye (0.1 mmol), nitromethane (27 μL, 0.5 mmol), solvent (0.5 mL), PSTA gelatin (2 mg), 37 °C, 6 h. [b] ^{1}H NMR yield. Diphenylmethane was used as the internal standard (second value was confirmed using DMA as the internal standard). Abbreviation: n.d. = not determined.

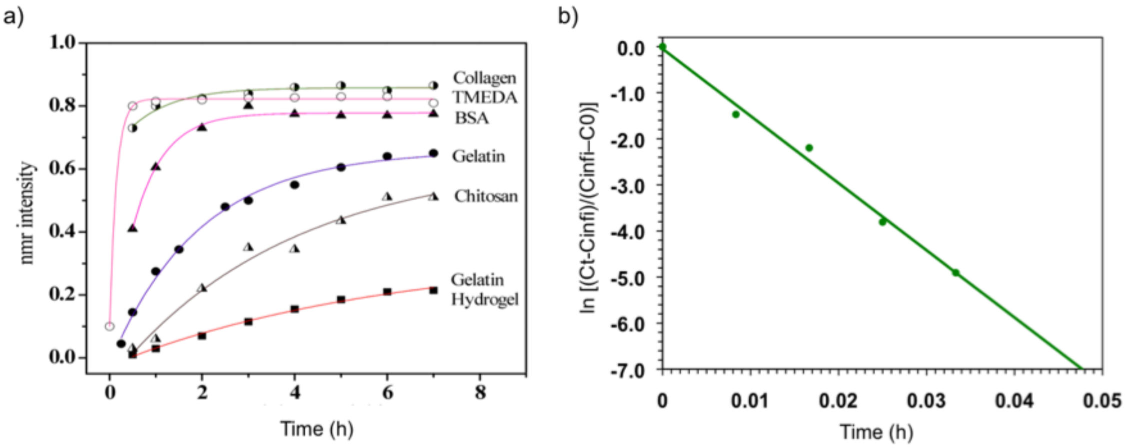

Figure S3: a) Typical evolution of reaction conversion over time, calculated by ^{1}H NMR analysis, for the Henry reaction catalyzed by different systems. b) Kinetic analysis of TMEDA-catalyzed Henry reaction: k = 158.22 ± 14.98 h^{-1}; R^2 = 0.9703.

5. Representative ^{1}H NMR spectra

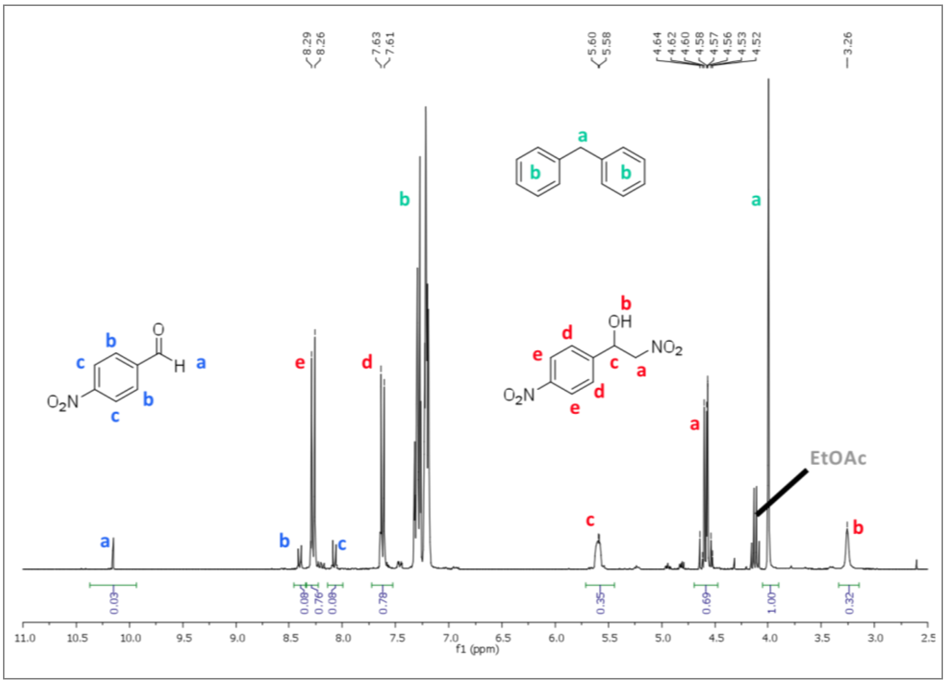

Figure S4: Representative crude ^{1}H NMR spectra of the gelatin-catalyzed Henry reaction between 4-nitrobenzaldehyde and nitromethane (5 equiv). Diphenylmethane was used as the internal standard.

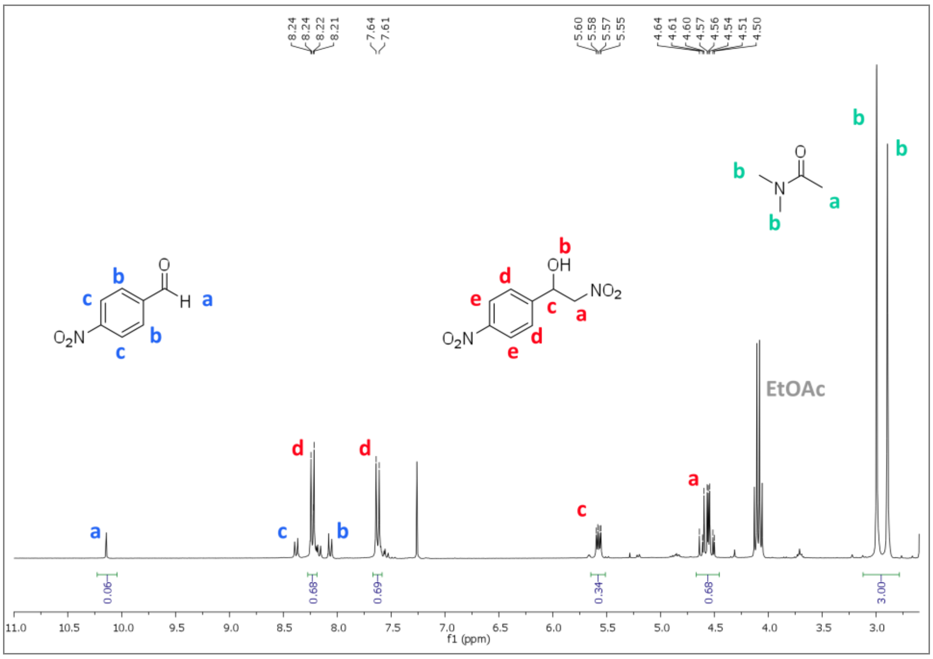

Figure S5: Representative crude ^{1}H NMR spectrum of the gelatin-catalyzed Henry reaction between 4-nitrobenzaldehyde and nitromethane (5 equiv). Dimethylacetamide was used as the internal standard (Note that methyl "a" of dimethylacetamide appears at ca. 2 ppm, overlapping with the signal from EtOAc).

6. Additional FESEM images

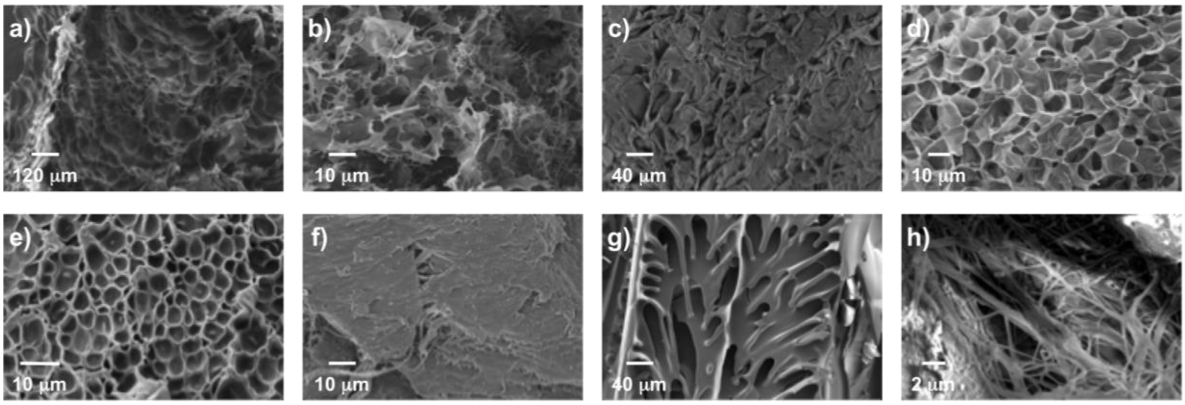

Figure S6: Additional FESEM images of different biocatalysts used in this work: a) esterificated PSTA gelatin; b) succinated PSTA gelatin; c-d) xerogel prepared by freeze-drying the hydrogel made of PSTA gelatin; e) cooked gelatin purchased from a supermarket; f) powdered chitosan; g) powdered BSA; h) powdered collagen.

7. References

1. L. C. R. Henry, *Hebd. Seances Acad. Sci.* **1895**, *120*, 1265–1268
2. D. Seebach, E. W. Colwin, F. Lehr, T. Weller, *Chimia* **1979**, *33*, 1–18.
3. a) C. Palomo, M. Oiarbide, A. Laso, *Eur. J. Org. Chem.* **2007**, 2561–2574; b) J. Boruwa, N. Gogoi, P. P. Saikia, N. C. Barua, *Tetrahedron: Asymmetry* **2006**, *17*, 3315–3326; c) C. Palomo, M. Oiarbide, A. Mielgo, *Angew. Chem. Int. Ed.* **2004**, *43*, 5442–5444; d) J. McNulty, J. Dyck, V. Larichev, A. Capretta, A. J. Robertson, *Lett. Org. Chem.* **2004**, *1*, 137–139.
4. a) J.-L. Wang, X. Li, H.-Y. Xie, B.-K. Liu, X.-F. Lin, *J. Biotechnol.* **2010**, *145*, 240–243, and references therein; b) K. R. Reddy, C. V. Rajasekhar, G. G. Krishna, *Synth. Commun.* **2007**, *37*, 1971–1976.
5. R. Ballini, G. Bosica, *J. Org. Chem.* **1997**, *62*, 425–427.
6. a) E. Busto, V. Gotor-Fernández, V. Gotor, *Org. Process Res. Dev.* **2011,** *15*, 236–240; b) M. López-Iglesias, E. Busto, V. Gotor, V. Gotor-Fernández, *Adv. Synth. Catal.* **2011**, *353*,

2345–2353.
7. K. K. Sharma, A. V. Biradar, T. Asefa, *ChemCatChem* **2010**, *2*, 61–66.
8. The isoelectric point is the pH at which a molecule or surface carries no net electrical charge. Hence, at a pH below their IEP, proteins carry a net positive charge, whereas above their IEP they carry a net negative charge.
9. Hence, in contrast to PSTA gelatin, the solution of BSTB gelatin could be above pH 7.5, which may mask the inherent catalysis of the Henry reaction by the protein.
10. Protein content of cold-water fish skin could not be obtained from the supplier.
11. R. Schrieber, H. Gareis, *Gelatine Handbook: Theory and Industrial Practice*, 1st Ed., Wiley-VCH Verlag GmbH, **2007**.
12. T. Ikeda, M. Tokita, A. Tsutsumi, K. Hikichi, *Jap. J. Appl. Phys.* **1990**, *29*, 353–355.
13. E. van den Bosch, C. Gielens, *Int. J. Biol. Macromol.* **2003**, *32*, 129–138.
14. For a comprehensive review on structure and properties of solid gelatin, see: P. V. Kozlov, G. I. Burdygina, *Polymer* **1983**, *24*, 651–666.
15. H. Firouzabadi, N. Iranpoor, A. Ghaderi, *Org. Biomol. Chem.* **2011**, *9*, 865–871.
16. C. V.-L. Bray, F. Jiang, X.-F. Wu, J.-B. Sortais, C. Darcel, *Tetrahedron Lett.* **2010**, *51*, 4555–4557.
